# Burnout and Workplace Bullying Among Teachers Across Educational Levels: A Cross-Sectional Study

**DOI:** 10.3390/ejihpe15120255

**Published:** 2025-12-11

**Authors:** António Portelada, Adelinda Candeias, Ana Lúcia João

**Affiliations:** 1 School of Education, Santarém Polytechnic University, 2001-902 Santarém, Portugal; 2 Comprehensive Health Research Center (CHRC), 7000-849 Évora, Portugal; aac@uevora.pt (A.C.); ana.joao@essaude.ipsantarem.pt (A.L.J.); 3 Health Sciences and Human Development, Évora University, 7000-849 Évora, Portugal; 4 Health Science School, Santarém Polytechnic University, 2005-075 Santarém, Portugal

**Keywords:** occupational burnout, workplace bullying, teachers, educational well-being, work environment

## Abstract

Burnout is a growing concern in the teaching profession, reflecting the inability to cope with persistent workplace stress and posing serious risks to teachers’ well-being and the sustainability of educational systems. It is characterised by diminished personal accomplishment, lack of fulfilment, and reduced productivity at work, usually expressed in three dimensions: emotional exhaustion, depersonalisation, and professional accomplishment. This study evaluated the relationship between burnout and workplace bullying among teachers in Portugal. Data were collected through an online questionnaire including sociodemographic information, the Maslach Burnout Inventory, and the Leymann Inventory of Psychological Terrorization scale (LIPT-60), with a final sample of 2003 teachers from preschool to higher education. Both instruments demonstrated excellent validity and reliability (KMO > 0.90; Cronbach’s α > 0.87). Most teachers presented a low risk of burnout (61.2%), while 36.9% were at moderate risk, with emotional exhaustion emerging as the most critical dimension (37.8% high levels). Statistically significant differences in burnout were found by gender, marital status, contractual relationship, working hours, and family separation. Workplace bullying correlated significantly with all burnout dimensions, with victims reporting higher emotional exhaustion and depersonalisation and lower professional accomplishment. These findings highlight the need for institutional strategies to reduce bullying and prevent teacher burnout, thereby promoting sustainable education systems.

## 1. Introduction

Teaching is a profession characterised by high emotional demands and complex interpersonal dynamics, which makes teachers particularly vulnerable to stress-related outcomes. Among these, burnout and workplace bullying have emerged as central concerns in educational research due to their impact on teacher well-being, professional engagement, and the overall functioning of schools. Although both constructs have been widely studied, less is known about how they interact across different educational levels within a large and diverse teacher population. Understanding this relationship is crucial, as the interplay between chronic stress and exposure to hostile behaviours may significantly compromise teachers’ psychological health and the sustainability of educational environments. This study therefore aims to analyse the association between burnout and workplace bullying among Portuguese teachers, offering comprehensive evidence from a large sample spanning preschool to higher education.

### 1.1. Burnout in the Teaching Profession

Burnout emerges as a gradual process in which individuals repeatedly try to manage persistent work-related demands without success. Conceptually, it is understood as a reaction to chronic occupational stress and comprises three core dimensions: emotional exhaustion, depersonalisation, and reduced professional accomplishment (Souza et al., 2016; Maslach & Leiter, 2016). Among these, reduced professional accomplishment refers to feelings of incompetence, lack of fulfilment, and diminished productivity at work (Maslach & Leiter, 2016).

Burnout affects a wide range of professions, including healthcare professionals, social workers, and security personnel, due to high emotional demands and constant pressure. This form of exhaustion is expressed through emotional fatigue, affective detachment, and loss of workplace efficacy (Sørengaard & Langvik, 2022; João et al., 2022; Nyamugoro et al., 2023; Carrión & Jiménez, 2023).

Education is a valued component across different cultures, and in this context, teaching is often perceived as an inherently rewarding profession (Agyapong et al., 2022). However, within the framework of educational restructuring and economic crises, this role, in addition to its rewards, brings significant challenges that adversely affect the working environment (Skaalvik & Skaalvik, 2017).

Teaching is therefore considered one of the professions most prone to burnout, due to frequent interaction with students, parents, and colleagues (Hakanen et al., 2006; Gomes et al., 2013; Calisto, 2016; Agyapong et al., 2022; Maratos et al., 2024). One of the critical issues faced by teachers in the workplace is burnout, which arises when they are pressured to perform duties that exceed their capacity or formal responsibilities (David & Quintão, 2012; Francisco et al., 2024). According to Maslach et al. (1997), teachers are increasingly expected to provide customer service-like support to address problems encountered daily by students and their families. Such responsibilities are often emotionally exhausting, as many of these problems have no immediate solution, thereby increasing stress levels and consequently contributing to burnout.

When professionals are unable to meet all demands, frustration may intensify, which in turn affects their energy (exhaustion), engagement (cynicism), and efficacy (Soini et al., 2016; Skaalvik & Skaalvik, 2020). In a study involving 333 Portuguese teachers, Gomes et al. (2013) found that 15% reported high levels of emotional exhaustion and 9% reported low professional accomplishment. These findings were attributed to excessive workloads and pressure to produce positive outcomes.

### 1.2. Workplace Bullying in Educational Settings

Workplace bullying, in turn, involves repetitive and systematic behaviours characterised by an asymmetrical power relationship and actions such as humiliation, manipulation, isolation, or disrespect, which generate feelings of threat or insecurity in victims, and may occur with or without explicit intent (Einarsen et al., 2011). This phenomenon tends to negatively affect victims, leading to physical and mental health issues, impairing organisational functioning, and producing broader social consequences (Carrión & Jiménez, 2023).

In a study conducted by Portelada et al. (2024) involving teachers, 22.5% identified themselves as victims of workplace bullying. This phenomenon was strongly associated with psychological distress, with 83% of victims reporting consequences such as anxiety, insomnia, and feelings of helplessness. The authors emphasised that the greater the frequency of hostile behaviours, the lower the teachers’ emotional well-being, self-esteem, and social engagement.

### 1.3. Relationship Between Burnout and Workplace Bullying

Ju et al. (2015) found that interpersonal relationships in the workplace are negatively associated with teacher burnout. This finding suggests that the greater the perceived support from supervisors and colleagues, the lower the incidence of burnout. Accordingly, the authors underscore the importance of promoting communication among peers and supervisors, thereby fostering a supportive school environment, which is crucial for job satisfaction and for protecting against burnout.

Workplace bullying appears to be a significant contributor to burnout among teachers, as highlighted in several studies (Mościcka-Teske et al., 2014; Bernotaite & Malinauskiene, 2017; Williams et al., 2023; Mukhtar et al., 2024). Hostile behaviours and persistent negative interactions within educational environments have been consistently associated with symptoms of professional burnout, such as emotional exhaustion and cynicism. These findings underscore the importance of implementing effective interventions aimed at promoting healthier work climates.

Research involving educators has shown that a proportion reported experiencing bullying to varying degrees, with a notable percentage also experiencing psychological distress (Bernotaite & Malinauskiene, 2017). Other studies have found that teachers subjected to workplace bullying often demonstrated increased emotional exhaustion and decreased professional efficacy (Mościcka-Teske et al., 2014). In higher education settings, the link between workplace bullying and burnout has also been observed, with emotional exhaustion and depersonalisation being particularly prominent among affected faculty members (Williams et al., 2023).

To address these challenges, scholars have emphasised the necessity of measures to reduce bullying and its negative consequences, particularly through the strengthening of interpersonal dynamics and support systems in schools (Mościcka-Teske et al., 2014; Bernotaite & Malinauskiene, 2017). Nevertheless, findings also indicate that not all educators experience severe burnout or bullying, pointing to the protective role of individual resilience and supportive organisational environments (Nwoko et al., 2023).

In this regard, institutions should foster workplace cultures that discourage harmful behaviours and promote professional relationships rooted in mutual respect and collaboration (Portelada et al., 2024). Moreover, schools are encouraged to implement structured programmes and training initiatives aimed at strengthening teacher resilience, improving interpersonal relationships, and promoting overall well-being among staff and their students (Candeias et al., 2023).

### 1.4. Study Objectives and Hypotheses

Given the above, the objective of the present study was to evaluate the relationship between burnout levels and workplace bullying among teachers in the Portuguese educational system. In line with this objective, the following research hypotheses were formulated:

**H1.**  *Higher levels of workplace bullying are positively associated with higher levels of emotional exhaustion among teachers.*

**H2.**  *Higher levels of workplace bullying are positively associated with higher levels of depersonalisation among teachers.*

**H3.**  *Higher levels of workplace bullying are negatively associated with professional accomplishment among teachers.*

**H4.**  *Teachers who perceive themselves as victims of workplace bullying present significantly higher levels of emotional exhaustion and depersonalisation, and lower levels of professional accomplishment, compared with those who do not consider themselves victims.*

**H5.**  *Sociodemographic and professional variables (gender, age, marital status, academic qualifications, teaching level, employment type, Schedule type, and family separation) are significantly associated with the dimensions of burnout.*

## 2. Materials and Methods

### 2.1. Study Design

This study is characterised as quantitative research, descriptive and correlational in nature, using a cross-sectional design. The main objective was to analyse the relationship between burnout and the sociodemographic and professional characteristics of teachers, as well as its association with workplace bullying.

### 2.2. Procedure

For data collection, an online questionnaire was developed using Google Forms. The instrument was organised into three parts. The first part included questions designed to obtain sociodemographic and professional information about the participating teachers. The second part contained a measure for assessing occupational burnout, namely the Maslach Burnout Inventory (MBI) mentioned previously. The third and final part included the Leymann Inventory of Psychological Terrorization scale (LIPT-60).

Confidentiality and anonymity of participants were safeguarded throughout. To ensure the full confidentiality of participants, the online questionnaire was designed so that no information enabling the direct or indirect identification of teachers was collected. Accordingly, no names, personal contacts, professional or institutional identification numbers, e-mail addresses, or any other sensitive data that could compromise anonymity were requested. Responses were automatically recorded in Google Forms without any association to personal accounts, and only general sociodemographic variables (e.g., age, gender, teaching level, type of contract) were collected, all presented in broad, non-identifiable categories. The data were stored in encrypted format and were accessible solely to the research team, ensuring that no individual information could be traced or disclosed. All procedures complied with the rules of the National Data Protection Commission and the applicable European legislation (GDPR).

The introduction to the questionnaire stated that the data collected would be used solely for research purposes and would not compromise the physical or psychological integrity of participants. It was also made clear that participants would not receive any financial compensation and could withdraw at any time without penalty.

Authorisation for the use of the questionnaire was obtained from the National Data Protection Commission and the Ethics Committee of the Portuguese Ministry of Health. Following this approval, school directors across Portugal were asked to disseminate the questionnaire online, and they collaborated in its distribution.

Finally, data processing and statistical analysis were carried out using the Statistical Package for the Social Sciences (SPSS) version 28.

### 2.3. Population and Sample

According to the most recent official data, there are approximately 208,310 teachers in Portugal (2023/24), covering preschool, basic, secondary, and higher education. Specifically, 17,642 teach in preschool, 31,579 in the first cycle of basic education, 22,279 in the second cycle, and 77,624 across the third cycle and secondary education. Meanwhile, the higher education sector comprises 41,361 teaching staff. Other sources (e.g., Info No. 8 from DGEEC) report nearly 189,999 educators in the preschool-to-secondary sector for 2022/23, of which 40,183 are in higher education (Direção-Geral de Estatísticas da Educação e Ciência, 2024a, 2024b; Conselho Nacional de Educação, 2023).

To ensure the adequacy of the sample, clear inclusion and exclusion criteria were established. Only teachers who were actively employed in Portuguese educational institutions at the time of data collection were eligible to participate. Educators who were retired, on medical leave, on parental or sabbatical leave, or not currently engaged in teaching activities were excluded from the study. Participation required the completion of the full online questionnaire and the provision of informed consent.

The recruitment followed a convenience sampling strategy, as participation was voluntary and based on self-selection. The questionnaire was disseminated nationally through multiple channels, including institutional e-mail sent to school directors, who were asked to forward the survey to their teaching staff, as well as professional networks, teacher associations, and informal dissemination via colleagues. No incentives were provided. Data were collected over a 2-month period, between January to February, allowing sufficient time for broad participation across educational levels.

Although the exact number of teachers who received the questionnaire could not be determined, due to secondary dissemination by schools and professional networks, it is estimated that the response rate was moderate given the total number of teachers in Portugal (approximately 208,310). A total of 2003 valid responses were obtained after applying the inclusion and exclusion criteria.

The online questionnaire was disseminated to the population of Portuguese teachers. A total of 2003 teachers responded, of whom 88.3% worked in public institutions. Regarding the level of education taught, 7.9% were preschool teachers, 46.4% primary school teachers, 28.2% secondary school teachers, and 17.4% higher education/university teachers.

Geographically, most participants worked in the central region of the country (41.2%). Only 24.2% worked in the north and 22.4% in the south.

Among the respondents, 76.9% were female, 66.4% were married or in a civil union, and their mean age was 47.73 years (SD = 8.20). With regard to academic and professional qualifications, 55.5% of teachers, i.e., more than half of the sample, held only a bachelor’s degree. Concerning higher academic degrees, 22.8% reported having a master’s degree and 13.1% a doctorate. However, the proportion of teachers with a specialty was low (4.4%).

At the institutional level, 69.8% of teachers held a permanent appointment, and 9.9% were employed within a regional placement framework, reflecting stable employment for most participants, with only 18% being contract teachers. Furthermore, 93.3% reported working full time.

In terms of distance from the workplace, 80.9% of the sample lived less than 30 km from their institution, and only 12.8% lived apart from their family.

### 2.4. Instruments

#### 2.4.1. Maslach Burnout Inventory

This study employed the Maslach Burnout Inventory, developed by Maslach et al. (1997), with validation studies conducted among Portuguese teachers. The second version of the MBI–Human Services Survey (MBI-HSS), previously validated for the Portuguese context by Martins (2008), was selected, as it provides a structure applicable across different educational settings and professional interactions. This choice was justified by the intention to assess teacher burnout not only in relation to students, but also in interactions with colleagues, supervisors, and other professionals within the school environment.

The MBI-HSS used in this study comprises 22 items distributed across three dimensions: emotional exhaustion (9 items), which assesses the capacity for emotional response in the professional context; depersonalisation (5 items), which analyses impersonal and detached attitudes towards work; and professional accomplishment (8 items), which measures perceived competence and efficacy in teaching practice. An example of an item is: “I feel emotionally drained by my work.”. Responses are rated on a seven-point Likert scale ranging from “never”, “a few times a year”, “once a month”, “a few times a month”, “once a week”, “a few times a week”, to “every day”, with participants asked to indicate the frequency with which they experienced the described situations.

#### 2.4.2. Leymann Inventory of Psychological Terrorization (LIPT-60)

In addition to the MBI, the LIPT-60 scale developed by Rivera and Abuín (2003) and validated among Portuguese teachers by Portelada et al. (2024), was used to assess workplace bullying. The LIPT-60 scale consists of 60 items that identify workplace bullying behaviours and consists of five response options ranging from 0 to 4, from “absolutely nothing” to “extremely”. An example of a typical item is: “Supervisors do not allow the person to express their views.” The scale comprises six factors: communication blockage and defamation, direct attacks, isolation, disregard for one’s work, personal discredit, and professional manipulation.

#### 2.4.3. Sociodemographic Questionnaire

A sociodemographic questionnaire was developed specifically for this study to characterise the personal and professional profile of participating teachers. It included closed-ended questions covering variables shown in the literature to be associated with burnout and workplace bullying. The variables assessed were age, gender, marital status, academic qualifications, teaching level, institution type (public or private), employment type, schedule type, and family separation due to professional duties. These variables were selected because they were used in the statistical analyses examining differences in burnout dimensions. All items were multiple-choice to ensure clarity, consistency, and ease of response.

### 2.5. Validity of the Instruments

The validity of factor analysis for the MBI and LIPT-60 scales was assessed using the Kaiser-Meyer-Olkin (KMO) measure of sampling adequacy. Values obtained were 0.913 for the MBI and 0.974 for the LIPT-60, both considered excellent. Bartlett’s test confirmed the suitability of factor analysis, with statistical significance observed (*p* < 0.001) for both scales.

In the MBI, three factors were identified corresponding to emotional exhaustion, professional accomplishment, and depersonalisation, which together explained 52.59% of the total variance. Internal consistency, assessed by Cronbach’s alpha, was 0.873, classified as very good.

In the LIPT-60, analysis revealed six factors, explaining 62.08% of the total variance. The identified dimensions were: “communication blockage and defamation”, “direct attacks”, “isolation”, “disregard for one’s work”, “personal discredit”, and “professional manipulation”. Cronbach’s alpha was 0.976, indicating excellent internal consistency.

### 2.6. Ethical Considerations

The authors of the LIPT-60 scale were contacted beforehand to grant formal permission for its use in the present study. After obtaining approval, the research protocol was submitted to the Ethics Committee of the Directorate-General for Education of the Ministry of Education, Science, and Innovation, which authorised the application of the scales under reference number 0512600001.

To ensure broad dissemination of the questionnaire, schools across all educational levels (preschool, primary, secondary, and higher education) were asked via email to distribute it, with additional encouragement for sharing among staff members to maximise participation.

All ethical principles were strictly observed, guaranteeing the confidentiality and anonymity of participants. In addition, all teachers provided written informed consent, ensuring compliance with research ethics guidelines.

### 2.7. Statistical Analysis

Descriptive statistics were used to characterise the sociodemographic profile of the sample and to examine the presence of chronic health conditions. For the inferential analyses, correlations between the MBI dimensions and the sociodemographic and professional characteristics of teachers were explored in order to identify factors associated with burnout and thereby deepen the understanding of its potential determinants. In addition, Pearson’s correlation coefficients were calculated to examine the relationships between the dimensions of burnout and workplace bullying.

Levels of burnout (low, moderate, and high) were classified according to the GEPEB criteria validated by Benevides-Pereira (2001), which follow the numerical cut-off scores established for the MBI-HSS subscales. The cut-off values used in this study were emotional exhaustion (EE): low ≤ 16, moderate = 17–26, high ≥ 27; depersonalisation (DP): low ≤ 6, moderate = 7–12, high ≥ 13; and professional accomplishment (PA): high ≥ 39, moderate = 32–38, low ≤ 31. These cut-offs are consistent with the normative recommendations of the MBI-HSS and have been used in previous Portuguese validation studies. Burnout risk levels were determined by combining these subscale classifications following the GEPEB model.

To ensure the adequacy of the parametric tests applied, the underlying statistical assumptions were examined prior to all inferential analyses. The normality of the distributions of the MBI dimensions and the LIPT-60 factors was assessed through histogram inspection, skewness and kurtosis coefficients, and the Kolmogorov–Smirnov test, which is recommended for large samples. Although the Kolmogorov–Smirnov test indicated statistical significance, this result was expected given the large sample size (*n* > 2000). Skewness and kurtosis values remained within acceptable limits, supporting the assumption of approximate normality for the use of parametric procedures.

The homogeneity of variances across groups was evaluated using Levene’s test. Whenever this assumption was violated, Welch’s correction, as implemented in SPSS, was applied for both independent samples *t*-tests and one-way ANOVA. Although the assumptions for parametric testing were generally met, alternative non-parametric procedures were defined *a priori* should violations occur. Specifically, the Mann–Whitney U test would replace the *t*-test, the Kruskal–Wallis test would substitute for ANOVA, and Spearman’s rho would be used instead of Pearson’s correlation coefficient. These procedures ensure the robustness of the analyses when distributional assumptions are not satisfied.

To complement the interpretation of statistical significance and to assess the magnitude of the observed effects, effect sizes were calculated for all inferential tests. Cohen’s *d* was reported for *t*-tests; eta squared (η^2^) was used for ANOVA and interpreted according to Cohen’s conventional thresholds (small ≥ 0.01, medium ≥ 0.06, large ≥ 0.14); and Pearson’s or Spearman’s *r* was reported for correlation analyses, interpreted as weak (0.10–0.29), moderate (0.30–0.49), or strong (≥0.50). These indices provided a more comprehensive understanding of the practical significance of the findings.

The dataset was examined for missing values prior to analysis. No systematic pattern of omissions was identified, and the proportion of missing data was minimal (<2%). Given this negligible level, complete case analysis (listwise deletion) was applied, as it did not compromise the robustness or representativeness of the statistical results.

All analyses were conducted using a 95% confidence level, with statistical significance set at *p* ≤ 0.05.

Data processing was performed using Microsoft Excel and SPSS (Statistical Package for the Social Sciences), version 28 for Windows.

## 3. Results

### 3.1. Burnout Among Teachers

Burnout risk was assessed using the criteria of the Research Group on Stress and Burnout (GEPEB), validated by Benevides-Pereira (2001). According to this criterion, high risk of burnout is identified when individuals present threshold values in two factors (high emotional exhaustion and depersonalisation, or low professional accomplishment). Moderate risk is identified when only one factor presents a threshold value. Conversely, teachers with medium or low levels of emotional exhaustion and depersonalisation and medium or high levels of professional accomplishment are classified as being at low risk of burnout.

Data analysis revealed that most participants (61.2%) presented low risk of burnout, while 36.9% were at moderate risk. Only 1.7% were at high risk, and 0.1% already exhibited clinical burnout (Table 1).

With respect to specific burnout dimensions, 53.6% of teachers presented medium scores and 37.8% high scores for emotional exhaustion. For professional accomplishment, the majority (82.8%) had high scores. Regarding depersonalisation, 74.9% presented low scores, 23.3% medium scores, and only 1.8% high scores (Table 1).

This section may be divided by subheadings. It should provide a concise and precise description of the experimental results, their interpretation, as well as the experimental conclusions that can be drawn.

### 3.2. Burnout and Sociodemographic and Professional Characteristics

Significant differences were observed in MBI dimensions of emotional exhaustion (*t*(2001) = −7.13, *p* = 0.001), professional accomplishment (*t*(762.9) = −3.46, *p* = 0.001), and depersonalisation (*t*(777.52) = 3.59, *p* = 0.001) in relation to gender (Table 2).

No significant differences were observed across all MBI dimensions regarding age and type of institution (Table 2).

With respect to marital status, statistically significant differences were found in emotional exhaustion (*F*(2;568.34) = 9.30, *p* = 0.001), professional accomplishment (*F*(2;571.19) = 5.00, *p* = 0.007), and depersonalisation (*F*(2;541.14) = 11.21, *p* = 0.001) (Table 2).

Regarding academic qualifications, significant differences were identified in emotional exhaustion (*F*(2;657.63) = 5.30, *p* = 0.005) and depersonalisation (*F*(2;3.85) = 4.38, *p* = 0.013) (Table 2).

At the level of teaching, statistically significant differences were observed in emotional exhaustion (*F*(3;21.46) = 9.81, *p* = 0.001) and depersonalisation (*F*(3;631.87) = 9.49, *p* = 0.001) (Table 2).

Institutional affiliation (*F*(2;14.10) = 6.42, *p* = 0.002) and working hours (*t*(1961) = 5.74, *p* = 0.001) were associated with statistically significant differences in emotional exhaustion (Table 2).

Finally, distance from family due to teaching responsibilities revealed significant differences in emotional exhaustion (*t*(350.87) = 3.77, *p* = 0.001), professional accomplishment (*t*(316.84) = −3.44, *p* = 0.001), and depersonalisation (*t*(311.41) = 4.94, *p* = 0.001).

With regard to gender, it was found that female teachers presented significantly higher mean values of emotional exhaustion (4.11 vs. 3.57) and professional accomplishment (5.59 vs. 5.39) compared with male teachers. However, in the dimension of depersonalisation, male teachers showed significantly higher mean values than female teachers (2.04 vs. 1.85).

Post hoc multiple comparison analysis using Tukey’s test, considering marital status, indicated significant differences in emotional exhaustion, professional accomplishment, and depersonalisation. For emotional exhaustion, single and divorced/separated teachers presented significantly higher mean values compared with married/civil union teachers (4.15 and 4.22 vs. 3.88). Regarding professional accomplishment, married teachers presented significantly higher mean values compared with single teachers (5.58 vs. 5.38). In depersonalisation, married/civil union teachers presented significantly lower mean values compared with divorced/separated and single teachers (1.83 vs. 2.01 and 2.08).

Using Tukey’s test for academic qualifications, statistically significant differences were observed in emotional exhaustion and depersonalisation. Teachers holding a bachelor’s degree presented higher mean values of emotional exhaustion compared with those holding a doctorate (4.04 vs. 3.73). Conversely, in depersonalisation, teachers with a doctorate showed higher mean values than those with a bachelor’s degree (2.00 vs. 1.84).

Post hoc analysis with Tukey’s test, considering level of education taught, revealed that primary school teachers presented significantly higher mean values of emotional exhaustion compared with higher education teachers (4.13 vs. 3.70), while secondary school teachers presented higher mean values compared with preschool teachers (3.97 vs. 3.65). Regarding depersonalisation, preschool teachers presented significantly lower mean values compared with teachers at the other levels of education (1.61 vs. 1.90, 1.92, and 1.96).

Teachers employed within the regional placement framework presented higher mean values of emotional exhaustion compared with teachers with permanent appointments and contracted teachers (4.28 vs. 3.97 and 3.81).

Teachers working full-time presented significantly higher mean values of emotional exhaustion compared with part-time teachers (4.02 vs. 3.25) (Table 2).

Teachers whose employment required separation from their families presented higher mean values of emotional exhaustion (4.28 vs. 3.98) and depersonalisation (2.20 vs. 1.85), and lower mean values of professional accomplishment (5.31 vs. 5.58), compared with teachers who worked close to their families.

In addition to the statistical significance values, the corresponding effect sizes were incorporated to provide a more comprehensive understanding of the magnitude and practical relevance of the observed differences. The gender comparisons revealed large effect sizes (Cohen’s *d* = 1.471 for emotional exhaustion, 1.052 for Personal Accomplishment, and 0.948 for depersonalisation), indicating substantial and meaningful distinctions between male and female teachers across all burnout dimensions. For the variables examined using ANOVA, namely age, marital status, academic qualifications, teaching level, and employment type, the effect sizes were generally small (*η*^2^ ranging from 0.001 to 0.016), suggesting that, despite some statistically significant group differences, these factors accounted for only a limited proportion of variance in burnout outcomes. By contrast, additional dichotomous comparisons revealed moderate to large effect sizes. Teachers working part-time, as well as those experiencing family separation due to professional obligations, displayed pronounced differences across all burnout dimensions (Cohen’s *d* values ranging from 1.052 to 1.485). The inclusion of these effect sizes therefore enhances the interpretation of the results by distinguishing between findings that are statistically significant and those that also carry substantive practical importance.

### 3.3. Burnout and Workplace Bullying

To assess correlations between MBI factors and LIPT-60 dimensions, Pearson’s correlation coefficients were calculated (Table 3).

The results show that the three MBI factors are significantly correlated with each other. Emotional exhaustion presents a negative and significant correlation with Personal Accomplishment (*r* = −0.141, *p* ≤ 0.01), indicating that higher levels of exhaustion are associated with a lower sense of accomplishment at work. On the other hand, emotional exhaustion is positively and moderately correlated with Depersonalization (*r* = 0.501, *p* ≤ 0.01), suggesting that as exhaustion increases, attitudes of distancing and indifference toward others also tend to rise. Personal Accomplishment, in turn, shows a negative and moderate correlation with Depersonalization (*r* = −0.321, *p* ≤ 0.01), indicating that lower levels of accomplishment are associated with higher levels of depersonalization. Overall, these results reflect the classic burnout pattern, in which exhaustion and depersonalization tend to intensify each other, while personal accomplishment decreases as the other dimensions worsen.

Emotional exhaustion was significantly correlated with “communication blockage and defamation” (*r* = 0.365), “isolation” (*r* = 0.213), “disregard for one’s work” (*r* = 0.356), and “personal discredit” (*r* = 0.202), with weak positive associations. It was also correlated with “direct attacks” (*r* = 0.157) and “professional manipulation” (*r* = 0.154), with very weak positive associations.

Professional accomplishment was significantly correlated, with very weak positive coefficients, with “communication blockage and defamation” (*r* = 0.105), “direct attacks” (*r* = 0.055), “isolation” (*r* = 0.096), “disregard for one’s work” (*r* = 0.101), and “personal discredit” (*r* = 0.056).

Depersonalisation presented a moderate positive correlation with “communication blockage and defamation” (*r* = 0.422), and weak positive associations with “direct attacks” (*r* = 0.280), “isolation” (*r* = 0.336), “disregard for one’s work” (*r* = 0.373), “personal discredit” (*r* = 0.311), and “professional manipulation” (*r* = 0.252).

### 3.4. Burnout and Awareness of Being a Victim of Workplace Bullying

When teachers were asked about their awareness of being victims of bullying and its association with MBI dimensions, statistically significant differences were found in emotional exhaustion (*t*(2001) = 13.04, *p* = 0.001), professional accomplishment (*t*(2001) = −3.34, *p* = 0.001), and depersonalisation (*t*(605.85) = 13.05, *p* = 0.001) (Table 4).

Teachers who reported awareness of being victims of workplace bullying presented significantly higher mean values of emotional exhaustion (4.74 vs. 3.75) and depersonalisation (2.46 vs. 1.73) compared with teachers who did not perceive themselves as victims. Conversely, in the dimension of professional accomplishment, teachers who considered themselves victims presented significantly lower mean scores (5.40 vs. 5.58)

## 4. Discussion

The psychometric analysis of the Maslach Burnout Inventory (MBI) conducted in this study reinforces the robustness of the instrument for assessing burnout in the Portuguese teaching population. The excellent sampling adequacy and the internal consistency of the three classical dimensions confirm that the MBI continues to capture the construct reliably in this professional group, in line with previous national validation studies. Importantly, these findings indicate that the patterns observed in the present research, namely the predominance of emotional exhaustion and the relative preservation of professional accomplishment, do not stem from measurement artefacts but reflect genuine tendencies within the teaching workforce. The confirmation of the three-factor structure also strengthens the interpretative validity of associations found between burnout and sociodemographic, professional, and relational variables. Similarly, the significant links between burnout and experiences of workplace bullying acquire greater empirical weight when supported by a psychometrically sound instrument, ensuring that the observed associations between emotional strain, depersonalisation, and exposure to hostile behaviours represent consistent and reliable phenomena. Overall, the validation evidence obtained in this study contributes to the credibility of the results and highlights the importance of using methodologically robust tools to monitor teacher well-being and burnout risk. These results confirm the validity and reliability of the MBI for assessing burnout among Portuguese teachers.

With respect to burnout prevalence, most teachers (61.2%) presented low risk, 36.9% were at moderate risk, and 1.7% were at high risk, with only 0.1% presenting fully developed burnout. These data suggest a reasonable capacity for adaptation among teachers, possibly sustained by coping strategies, social support, and professional experience. However, the proportion of teachers at moderate or high risk warrants attention, as these groups are particularly vulnerable to progression of burnout if preventive and intervention measures are not implemented. Previous studies indicate that the school environment, especially in contexts of work overload and limited recognition, fosters the emergence of burnout symptoms (David & Quintão, 2012; Francisco et al., 2024). Additional evidence also highlights the role of excessive workload in increasing burnout risk among teachers (Agyapong et al., 2024).

In analysing burnout dimensions, more than half of teachers (53.6%) reported medium levels of emotional exhaustion, and 37.8% reported high levels, confirming that this is the most compromised dimension. This exhaustion may be a consequence of heavy workloads, pressure for results, and challenges in managing large and heterogeneous classes, as emphasised by Skaalvik and Skaalvik (2020), who identified exhaustion as the initial manifestation of teacher burnout. On the other hand, professional accomplishment was the most preserved dimension, with 82.8% of participants reporting high scores, suggesting that despite strain, many teachers continue to derive meaning and satisfaction from their professional practice, with this dimension functioning as a protective factor (Maratos et al., 2024). Depersonalisation showed very low prevalence (1.8%), indicating that teachers generally maintain an affective connection with their students and resort little to emotional distancing strategies, consistent with the findings of Hofmann et al. (2023).

Regarding sociodemographic variables, statistically significant differences were observed in all three burnout dimensions by gender: women presented higher emotional exhaustion and higher professional accomplishment, whereas men presented higher depersonalisation, corroborating the studies of Carlotto (2011), Ferradás et al. (2019), Kollerová et al. (2023) and Agyapong et al. (2024). These results may reflect greater burdens assumed by women, particularly in balancing professional and family life, as well as a stronger emotional attachment to teaching. Conversely, higher depersonalisation among men may relate to defensive strategies more characteristic of traditionally masculine roles, such as affective detachment.

Concerning marital status, single or divorced teachers presented higher levels of emotional exhaustion and depersonalisation, similar to the findings of Stathopoulou et al. (2023), which may be explained by a weaker social support network outside the professional context. By contrast, married teachers or those in civil unions reported higher levels of professional accomplishment, reinforcing the importance of emotional support as a protective factor, as demonstrated by Turner et al. (2022) and Muntean et al. (2022).

With respect to academic qualifications, teachers with a bachelor’s degree presented higher emotional exhaustion, possibly due to lower professional recognition or fewer career progression opportunities. Interestingly, teachers with doctorates, although less exhausted, presented higher levels of depersonalisation, which may reflect a misalignment between expectations and professional reality. Literature has reported contradictory findings (Chryssouli & Koutroukis, 2023; Pakdee et al., 2025), suggesting that the relationship between academic qualifications and burnout is complex.

Teaching level also showed an impact: primary and secondary teachers presented higher emotional exhaustion than higher education and preschool teachers, consistent with the findings of Ozamiz-Etxebarria et al. (2023). This may be due to the specific pedagogical and behavioural demands of these levels, as well as increased administrative workload. Depersonalisation was lower among preschool teachers, possibly due to closer affective relationships with pupils, which is characteristic of this stage. The findings of Agyapong et al. (2024) suggest that environmental pressures, such as larger class sizes, higher behavioural demands, and greater organisational complexity, may intensify emotional strain.

Employment conditions also influenced burnout: teachers within the regional placement framework presented higher emotional exhaustion compared with those with permanent or contracted positions, possibly due to the instability associated with that placement. Full-time schedules were also associated with higher emotional exhaustion, confirming the direct relationship between workload and burnout risk, as reported by Williams et al. (2023). Moreover, separation from family proved to be a vulnerability factor: teachers in this situation reported higher emotional exhaustion, higher depersonalisation, and lower professional accomplishment, in line with studies emphasising the negative impact of prolonged family separation on teacher well-being (Li et al., 2024).

Regarding the relationship between burnout and workplace bullying, data showed statistically significant correlations between all MBI dimensions and the various forms of bullying assessed by the LIPT-60 scale. Emotional exhaustion presented moderate correlations with “communication obstruction and defamation” and “professional discredit”, indicating that interpersonal conflict and professional devaluation exacerbate exhaustion, as also demonstrated by Kollerová et al. (2023) and Mościcka-Teske et al. (2014). Depersonalisation was also strongly correlated with these forms of bullying, suggesting the adoption of emotional distancing strategies as a response to hostile environments, consistent with Bernotaite and Malinauskiene (2017). Professional accomplishment presented weak correlations with bullying factors, which may indicate that despite exposure to psychological violence, some teachers maintain a positive perception of efficacy and professional value, a phenomenon already described in studies on teacher resilience (Ferradás et al., 2019; Agyapong et al., 2022).

It should also be noted that teachers who reported awareness of being victims of bullying presented significantly higher levels of emotional exhaustion and depersonalisation, and lower levels of professional accomplishment. This finding is particularly concerning as it reveals the direct impact of workplace bullying on the development of burnout, confirming empirical evidence linking psychological violence at work to increased emotional exhaustion, cynicism, and demotivation among teachers (Mościcka-Teske et al., 2014; Bernotaite & Malinauskiene, 2017; Williams et al., 2023; Mukhtar et al., 2024).

After discussing the main findings in light of the existing literature, it is important to examine how the results relate to the hypotheses formulated in this study. Regarding the research hypotheses, all assumptions were supported by the data. H1 and H2 were confirmed, as higher levels of workplace bullying were associated with higher emotional exhaustion and depersonalisation. H3 was not supported, with bullying showing a positive relationship with professional accomplishment. H4 was confirmed since teachers who perceived themselves as victims of bullying presented significantly higher emotional exhaustion and depersonalisation and lower professional accomplishment than those who did not. Finally, H5 partially supported, as several sociodemographic and professional variables, such as gender, marital status, academic qualifications, teaching level, employment type, working hours, and family separation, showed significant associations with burnout dimensions.

Overall, this study contributes to the contemporary literature by providing one of the most recent large-scale analyses of burnout and workplace bullying among Portuguese teachers. The results identify clear risk profiles, emphasise the importance of workplace relational climate, and reinforce the relevance of using psychometrically sound instruments in monitoring teacher well-being. From an applied perspective, the findings suggest the need for reducing workload, strengthening organisational support, promoting respectful professional relationships and implementing early detection strategies for burnout symptoms.

### Limitations

Although the present study provides relevant findings on teacher burnout and its relationship with workplace bullying, certain methodological limitations must be acknowledged that may influence the interpretation and generalisation of the data.

Firstly, the cross-sectional design precludes the identification of causal relationships between the variables analysed. Thus, although statistically significant associations were observed between burnout and factors such as bullying and certain sociodemographic characteristics, it is not possible to ascertain whether these are causes or consequences of burnout.

Secondly, the use of an online self-report questionnaire, while enabling access to a large number of participants and ensuring anonymity, may have introduced social desirability bias, as well as subjective interpretations of questions and variations in item comprehension. Furthermore, data on bullying were based on individual perceptions, which may not objectively reflect the occurrence of such behaviours.

The sample was obtained through convenience and voluntary online participation, which represents a limitation regarding the representativeness of the Portuguese teaching population. It is possible that teachers more sensitive to the topic, or those experiencing greater psychological distress, were more motivated to participate, introducing potential selection bias.

There was also an overrepresentation of female teachers (76.9%) and those working in public schools (88.3%), which may limit the generalisation of results to male teachers or those working in private institutions. This imbalance also complicates more robust subgroup comparisons.

Another important limitation concerns the composition of the sample. Female teachers and those working in public institutions were substantially overrepresented, which restricts the generalisability of the findings to male teachers and those employed in private schools. This imbalance also reduces the robustness of subgroup comparisons and may obscure potential gender, or sector specific patterns. Future studies should therefore adopt sampling strategies aimed at increasing representativeness, such as stratified sampling across gender and institution type, targeted recruitment in private schools, and the use of proportional quotas. Additionally, combining online recruitment with direct institutional engagement may help reduce volunteer bias and ensure more balanced participation across demographic and organisational subgroups.

Finally, the absence of contextual and institutional variables such as actual workload, number of classes, psychosocial support structures, leadership styles, or institutional disciplinary history should be highlighted. Inclusion of such variables could enrich the analysis and allow a deeper understanding of the organisational conditions influencing burnout.

## 5. Conclusions

This study examined, in a large sample of Portuguese teachers, the relationship between burnout and workplace bullying, showing that although most participants presented a low risk of burnout, a considerable proportion were at moderate risk and therefore vulnerable to the progression of the syndrome. Emotional exhaustion was the most affected dimension, suggesting that accumulated strain remains a structural challenge within the teaching profession.

The differences identified across sociodemographic and professional groups reinforce that burnout is not a homogeneous phenomenon; rather, it is shaped by working conditions, contractual stability, workload, educational level, and family support. These findings are consistent with literature indicating that the work context and emotional demands of teaching are central determinants of professional well-being.

Importantly, workplace bullying showed significant associations with all burnout dimensions, particularly emotional exhaustion and depersonalisation. Furthermore, teachers who perceived themselves as victims of hostile behaviours reported higher levels of burnout, underscoring the immediate and detrimental impact of these experiences. These results add to a growing body of evidence identifying adverse relational climates as a critical factor in the erosion of teachers’ psychological health.

Although the cross-sectional design limits causal inference and the non-probabilistic sampling restrict the generalisability of results, the study offers meaningful contributions by demonstrating, using psychometrically robust instruments, that teacher burnout is closely linked to schools’ relational and organisational dynamics.

It is therefore imperative that educational institutions invest in preventive policies that promote healthy working environments, reduce bullying practices, and strengthen social and emotional support systems. Strategies such as training in conflict management, fostering empathetic leadership, psychological support services, and programmes that enhance resilience and professional efficacy may constitute effective measures to mitigate burnout.

In summary, the findings of this study provide strong evidence that promoting teacher well-being is essential for the sustainability of educational systems. Preventing burnout and workplace bullying is not only an organisational responsibility but also a prerequisite for ensuring educational contexts that are fairer, healthier, and more conducive to high-quality teaching.

## Figures and Tables

**Table 1:** Prevalence of burnout and MBI dimension scores.

**Burnout Risk and Prevalence**		***n***	**%**
Low Burnout Risk		1226	61.2
Moderate Burnout Risk		740	36.9
High Burnout Risk		35	1.7
Burnout Present		2	0.1
**Burnout dimensions**		***n***	**%**
Emotional Exhaustion	Low	171	8.5
	Moderate	1074	53.6
	High	758	37.8
Personal Accomplishment	Low	21	1.0
	Moderate	324	16.2
	High	1658	82.8
Depersonalisation	Low	1500	74.9
	Moderate	466	23.3
	High	37	1.8

**Table 2:** Significance of MBI dimension differences by sociodemographic and professional variables.

Variables		Emotional Exhaustion	Personal Accomplishment	Depersonalisation
		M (DP)	M (DP)	M (DP)
Gender	Male	3.57 (1.47)	5.39 (1.18)	2.04 (1.04)
	Female	4.11 (1.47)	5.59 (1.01)	1.85 (0.91)
	*Sig.*	0.001 ***	0.001 ***	0.001 ***
	*d*	1.471	1.052	0.948
Age	20–30	3.98 (1.40)	5.13 (1.34)	1.94 (1.12)
	31–40	4.02 (1.39)	5.55 (1.00)	1.95 (0.93)
	41–50	4.04 (1.45)	5.59 (1.02)	1.86 (0.91)
	51–60	3.92 (1.54)	5.51 (1.11)	1.89 (0.99)
	>60	3.52 (1.73)	5.58 (0.98)	1.84 (0.85)
	*Sig.*	0.115	0.249	0.609
	*η* ^2^	0.007	0.004	0.001
Marital Status	Single	4.15 (1.52)	5.38 (1.11)	2.08 (1.08)
	Married	3.88 (1.47)	5.58 (1.04)	1.83 (0.90)
	Divorced/Widowed	4.22 (1.49)	5.55 (1.02)	2.01 (0.99)
	*Sig.*	0.001 ***	0.007 **	0.001 ***
	*η* ^2^	0.009	0.005	0.014
Academic Qualifications	Bachelor’s Degree	4.04 (1.51)	5.56 (1.04)	1.84 (0.91)
	Master’s Degree	3.96 (1.49)	5.46 (1.11)	1.95 (1.00)
	PhD	3.73 (1.37)	5.57 (1.07)	2.00 (0.96)
	*Sig.*	0.005 ***	0.237	0.013 *
	*η* ^2^	0.006	0.003	0.006
Teaching Level	Preschool education	3.65 (1.34)	5.71 (0.95)	1.61 (0.68)
	Elementary education	4.13 (1.47)	5.57 (1.02)	1.90 (0.96)
	Secondary education	3.97 (1.55)	5.47 (1.12)	1.92 (1.01)
	Higher education	3.70 (1.43)	5.51 (1.08)	1.96 (0.92)
	*Sig.*	0.001 ***	0.062	0.001 ***
	*η* ^2^	0.016	0.004	0.008
Institution Type	Public	3.98 (1.49)	5.55 (1.05)	1.89 (0.95)
	Private	3.89 (1.51)	5.51 (1.11)	1.92 (0.96)
	*Sig.*	0.191	0.318	0.339
	*d*	1.489	1.056	0.951
Employment Type	Permanent contract	3.97 (1.49)	5.58 (1.03)	1.88 (0.95)
	Pedagogical zone contract	4.28 (1.49)	5.37 (1.20)	1.98 (1.02)
	Fixed-term	3.81 (1.44)	5.50 (1.07)	1.89 (0.94)
	*Sig.*	0.002 **	0.052	0.417
	*η^2^*	0.005	0.004	0.001
Schedule type	Full-time	4.02 (1.48)	5.55 (1.05)	1.90 (0.95)
	Part-time	3.25 (1.47)	5.46 (1.09)	1.81 (0.90)
	*Sig.*	0.001 ***	0.173	0.139
	*d*	1.477	1.056	0.951
Family Separation	Yes	4.28 (1.38)	5.31 (1.18)	2.20 (1.09)
	No	3.93 (1.50)	5.58 (1.03)	1.85 (0.92)
	*Sig.*	0.001 ***	0.001 ***	0.001 ***
	*d*	1.485	1.052	0.944

* *p* ≤ 0.05; ** *p* ≤ 0.01; *** *p* ≤ 0.001; *d* = Cohen’s d; *η*^2^ = eta squared.

**Table 3:** Correlations between MBI and LIPT-60 dimensions.

Dimensions	MBI—Emotional Exhaustion	MBI—Professional Accomplishment	MBI—Depersonalisation
MBI—Emotional Exhaustion	1	−0.141 **	0.501 **
MBI—Professional Accomplishment	−0.141 **	1	−0.321 **
MBI—Depersonalisation	0.501 **	−0.321 **	1
LIPT-60—Communication Blockage and Defamation	0.365 **	0.105 **	0.422 **
LIPT-60—Direct Attacks	0.157 **	0.055 *	0.280 **
LIPT-60—Isolation	0.213 **	0.096 **	0.336 **
LIPT-60—Disregard for One’s Work	0.356 **	0.101 **	0.373 **
LIPT-60—Personal Discredit	0.202 **	0.056 *	0.311 **
LIPT-60—Professional Manipulation	0.154 **	0.027	0.252 **

* *p* ≤ 0.05; ** *p* ≤ 0.01.

**Table 4:** Significance of differences in MBI dimensions according to awareness of being a victim of workplace bullying.

Awareness of Being a Victim of Bullying	Yes		No		Sig.
	M	DP	M	DP	
Emotional Exhaustion	4.74	1.44	3.75	1.43	0.001 ***
Professional Accomplishment	5.40	1.06	5.58	1.05	0.001 ***
Depersonalisation	2.46	1.10	1.73	0.83	0.001 ***

*** *p* ≤ 0.001.

## Data Availability

Due to ethical issues, the data collected and analysed in this study are not available to outside researchers.
